# Outbreak of OXA-48-producing *Enterobacteriaceae* in a neonatal intensive care unit in Western Sweden

**DOI:** 10.1007/s10096-023-04584-y

**Published:** 2023-03-20

**Authors:** Erika Tång Hallbäck, Anna Johnning, Sofia Myhrman, Marie Studahl, Elisabet Hentz, Anders Elfvin, Ingegerd Adlerberth

**Affiliations:** 1 Department of Infectious Diseases, Institute of Biomedicine, Sahlgrenska Academy, University of Gothenburg, Gothenburg, Sweden; 2 Department of Clinical Microbiology, Sahlgrenska University Hospital, Region Västra Götaland, Gothenburg, Sweden; 3 Department of Mathematical Sciences, Chalmers University of Technology and University of Gothenburg, Gothenburg, Sweden; 4 Department of Systems and Data Analysis, Fraunhofer-Chalmers Centre, Gothenburg, Sweden; 5 Centre for Antibiotic Resistance Research in Gothenburg (CARe), Gothenburg, Sweden; 6 Department of Clinical Microbiology, Infection Control Unit, Sahlgrenska University Hospital, Region Västra Götaland, Gothenburg, Sweden; 7 Department of Infectious Diseases, Sahlgrenska University Hospital, Region Västra Götaland, Gothenburg, Sweden; 8 Department of Pediatrics, Institute of Clinical Sciences, Sahlgrenska Academy, University of Gothenburg, Gothenburg, Sweden; 9 Department of Pediatrics, The Queen Silvia Children’s Hospital, Sahlgrenska University Hospital, Region Västra Götaland, Gothenburg, Sweden

**Keywords:** *Enterobacteriaceae*, OXA-48, Antibiotic resistance, Whole-genome sequencing, New-born infant, Neonatal intensive care, Outbreak, Plasmid transmission

## Abstract

In 2015, an outbreak caused by OXA-48-producing *Enterobacteriaceae* affected a neonatal intensive care unit at a Swedish University Hospital. The aim was to explore the transmission of OXA-48-producing strains between infants and the transfer of resistance plasmids between strains during the outbreak. Twenty-four outbreak isolates from ten suspected cases were whole-genome sequenced. A complete assembly was created for the index isolate (*Enterobacter cloacae*) and used as a mapping reference to detect its plasmids in the remaining isolates (17 *Klebsiella pneumoniae*, 4 *Klebsiella aerogenes*, and 2 *Escherichia coli*). Strain typing was performed using core genome MLST and SNP analysis. As judged from sequencing and clinical epidemiological data, the outbreak involved nine cases (two developed sepsis) and four OXA-48-producing strains: *E. cloacae* ST1584 (index case), *K. pneumoniae* ST25 (eight cases), *K. aerogenes* ST93 (two cases), and *E. coli* ST453 (2 cases). Two plasmids from the index strain, p*Ecl*A2 and p*Ecl*A4, carrying *bla*_OXA48_ and *bla*_CMY-4_, respectively, were traced to all *K. pneumoniae* ST25 isolates. *Klebsiella aerogenes* ST93 and *E. coli* ST453 harboured either only p*Ecl*A2, or both p*Ecl*A2 and p*Ecl*A4. One suspected case harbouring OXA-162-producing *K. pneumoniae* ST37 could be excluded from the outbreak. Once initiated by an *E. cloacae* strain, the outbreak was caused by the dissemination of a *K. pneumoniae* ST25 strain and involved inter-species horizontal transfer of two resistance plasmids, one of which carried *bla*_OXA-48_. To our knowledge, this is the first description of an outbreak of OXA-48-producing *Enterobacteriaceae* in a neonatal setting in northern Europe.

**Supplementary Information:**

The online version contains supplementary material available at 10.1007/s10096-023-04584-y.

## Introduction

During the last decades, there has been a worrying rise in Gram-negative intestinal bacteria producing carbapenemases, i.e. b-lactamases that hydrolyse carbapenems and usually also other b-lactam antibiotics. These bacteria are particularly troubling since they are often resistant to several additional classes of antibiotics which further limits available treatment options. The incidence of carbapenem-producing *Enterobacteriaceae* is low but increasing in Sweden: from 0.2 to 1.3 cases/100,000 inhabitants between 2012 and 2021 [1]. Some of the most frequently encountered carbapenemases are those of the OXA-48 group [2]. These enzymes hydrolyse carbapenems only at a low level and show weak activity against cephalosporins, but high-level resistance to carbapenems can be achieved when combined with permeability defects and/or co-expression of extended-spectrum b-lactamases (ESBLs) or AmpC b-lactamases [3, 4]. Carbapenemases of the OXA-48 group, hence, constitute a threat to our ability to treat bacterial infections and it is, therefore, important to understand how *bla*_OXA-48-like_ genes are disseminated in bacterial populations.

Neonatal care units (NICUs) frequently experience spread of *Enterobacteriaceae* [5] and outbreaks involving OXA-48-producing *Enterobacteriaceae* strains in this setting have been reported [6–8]. Due to sparse colonization by anaerobic bacteria during the first weeks of life, members of the *Enterobacteriaceae* family establish and reach high abundance levels in the neonatal gut. As the gut microbiota matures, the proliferation of *Enterobacteriaceae* and other facultative anaerobic bacteria is suppressed [9]. The acquisition of anaerobes is severely delayed in preterm infants, partly related to comprehensive antibiotic treatments, rendering this population prone to heavy colonization by *Enterobacteriaceae* [10]. A high concentration of these bacteria in the gut lumen may favour bacterial conjugation and plasmid transfer, which requires close contact between bacterial cells[11–13]. The intestines of preterm infants could therefore represent a hotspot for horizontal transfer of *bla*_OXA-48_ and other plasmid-borne resistance genes, as also indicated by a recent experimental study [14].

In this study, we describe an outbreak in a NICU at a Swedish University Hospital. The outbreak involved ten suspected cases and four different OXA-48-producing *Enterobacteriaceae* species: *Enterobacter cloacae*, *Klebsiella pneumoniae*, *Klebsiella aerogenes*, and *Escherichia coli*. We used whole-genome sequencing to identify both clonal spread between cases and the dissemination of plasmids, originating in the index strain (*E. cloacae*), to other strains. The results strongly indicate horizontal transfer in vivo—between both species and genera—of two different plasmids encoding the carbapenemase OXA-48 and the AmpC-type b-lactamase CMY-4, respectively.

## Materials and methods

### The setting, data collection, and definitions

The outbreak occurred in 2015 in an NICU of a tertiary care University Hospital in Western Sweden. Comprised of 14 beds allocated to six rooms, the ward provides specialist care for approximately 1000 term and preterm infants (born from gestational week 22 + 0) annually. In 2015, rectal screening cultures were obtained from all patients at admission to the ward and weekly thereafter. If carriage of ESBL- or carbapenemase-producing bacteria was suspected or confirmed by the Clinical Microbiology laboratory, the patient was cared for in a single-bed room by a dedicated nurse.

**Fig. 1:**
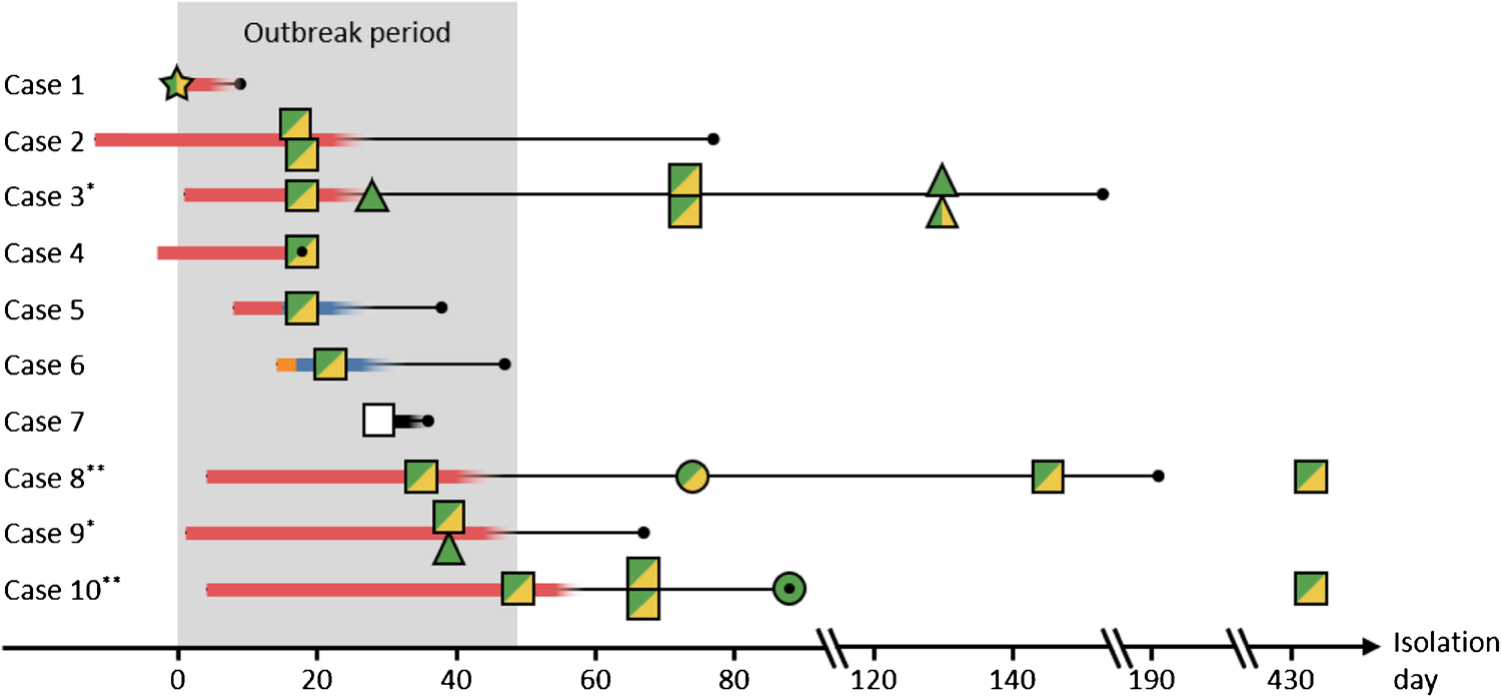
Timeline of the outbreak with OXA-48-producing *Enterobacteriaceae* in a neonatal unit. Isolation day, species identity and carriage of the outbreak plasmids are shown for the isolates selected from the ten suspected cases, with the index isolate (Case 1) set as day 0. Twin pair one (*) and twin pair two (**) are marked with asterisks. Horizontal lines represent the duration of hospital care, and their colour indicates the ward caring for each case until case identification: red = Ward A, blue = Ward B, black = Ward C, orange = Ward D. Symbols represent species: star = *E. cloacae*, square = *K. pneumoniae*, triangle = *K. aerogenes*, and circle = *E. coli*. Carriage of outbreak plasmids is indicated by symbol colour: green = carriage of *pEclA2*, yellow = carriage of *pEclA4*, white = no carriage of outbreak plasmids

Details on contact tracing and sampling were retrospectively collected from the patient charts and reports from the infection prevention and control team. A suspected case was defined as a patient within the NICU from whom an *Enterobacteriaceae* strain positive for carbapenemases of the OXA-48 group was isolated. The outbreak period was defined from the identification of the first suspected case—i.e. the sampling date of the case’s first positive isolate—to the identification of the last suspected case. No additional infants colonized or infected by *Enterobacteriaceae* positive for carbapenemases of the OXA-48-group were identified at the NICU past this period within the following year. (Fig. 1, Supplementary Fig. S1).

The study was approved by the Regional Ethical Review Board in Gothenburg, Sweden (no. 997–18 and no. 228–15).

### Culturing, bacterial identification and characterization, and selection of isolates

During the outbreak period, ten infants were colonized and/or infected by *Enterobacteriaceae* positive for OXA-48-like carbapenemases. The methods used for culturing, bacterial identification, antimicrobial susceptibility testing, and identification of resistance determinants were those routinely applied in the clinical microbiology laboratory at the University Hospital (Supplementary Material). A total of 155 rectal screening samples were obtained from the ten suspected outbreak cases during and after the outbreak period, of which 78 were assessed as positive for *Enterobacteriaceae* bacteria producing carbapenemases of the OXA-48 group (Supplementary Figure S1). In addition, four clinical samples—from two of the suspected cases (two blood cultures, two nasopharyngeal cultures) —were positive for such bacteria (Supplementary Figure S1). In total, 116 isolates assessed as positive for carbapenemases of the OXA-48 group were obtained from the 82 positive samples and 74/116 isolates were saved and stored frozen (− 80 °C) at the Clinical Microbiology laboratory.

For this study, we selected 24 of the stored isolates with confirmed carriage of genes encoding a carbapenemase of the OXA-48 group. For each suspected case, the first identified isolate of each OXA-48-positive species colonizing that infant was selected (*n* = 14). For Case 2, the first identified isolate was a blood culture isolate, and both the blood isolate and the rectal screening isolate from the following day were selected for analysis. Furthermore, from three persistently colonized cases, nine isolates were selected that showed deviations in phenotypic resistance tests and/or meropenem MIC-values from previous isolates of that species in the colonized infant. In total, 1 *E. cloacae*, 17 K*. pneumoniae*, 4 K*. aerogenes*, and 2 *E. coli* isolates were selected for whole-genome sequencing. (Table 1, Supplementary Figure S1).

**Table 1:** Isolates positive for carbapenemases of the OXA-48 group selected for whole-genome sequencing

Isolate ID ^a^	Case	Species	Location	Isolation day ^b^	Ward ^c^	CCUG no	NCBI accession no	MIC ^d^	Resistance phenotypes ^e^									β-lactamases ^f^	
								MP	CT	CAZ	MP	TZP	TM	CI	TS	IP	CO	ESBL	AmpC
*Ecl*1A*	1	*E. cloacae*	Faeces	0	A	67,822	JAGPNS000000000	4	R	R	I	R	I	R	R	I	S	0	–
*Kpn*2A1	2	*K. pneumoniae*	Blood	17	A	67,824	JAGPNT000000000	0.125	R	R	S	R	S	S	R	–	–	1	1
*Kpn*2A2	2	*K. pneumoniae*	Faeces	18	A	67,823	JAGPNU000000000	1	R	R	S	R	S	S	R	–	S	1	1
*Kpn*3A1	3	*K. pneumoniae*	Faeces	18	A	75,156	JAGPNV000000000	2	R	R	S	R	S	S	R	–	S	1	–
*Kae*3A2	3	*K. aerogenes*	Faeces	28	A	67,825	JAGPNW000000000	32	R	I	R	R	S	S	S	R	–	–	–
*Kpn*3B3	3	*K. pneumoniae*	Faeces	73	B	75,157	JAGPNX000000000	32	R	R	R	R	S	S	R	–	–	–	1
*Kpn*3B4	3	*K. pneumoniae*	Faeces	73	B	75,158	JAGPNY000000000	2	R	R	S	R	S	S	R	–	–	1	1
*Kae*3D5	3	*K. aerogenes*	Faeces	130	D	75,159	JAGPNZ000000000	8	I	S	I	R	S	S	S	I	S	1	1
*Kae*3D6	3	*K. aerogenes*	Faeces	130	D	75,160	JAGPOA000000000	4	R	R	I	R	S	S	S	I	S	1	1
*Kpn*4A	4	*K. pneumoniae*	Faeces	18	A	75,168	JAGPOB000000000	1	R	R	S	R	S	S	R	–	–	1	1
*Kpn*5B	5	*K. pneumoniae*	Faeces	18	B	67,830	JAGPOC000000000	0.25	R	R	S	R	S	S	R	–	–	1	1
*Kpn*6A	6	*K. pneumoniae*	Faeces	22	B	67,831	JAGPOD000000000	1	R	R	S	R	S	S	S	S	S	1	1
*Kpn*7C	7	*K. pneumoniae*	Faeces	29	C	74,849	JAGPOE000000000	2	R	R	I	R	S	S	S	S	–	1	–
*Kpn*8A1	8	*K. pneumoniae*	Faeces	35	A	67,826	JAGPOF000000000	1	R	R	S	R	S	S	R	–	–	1	1
*Eco*8A2	8	*E. coli*	Faeces	74	A	75,163	JAGPOG000000000	0.5	R	R	S	R	S	S	S	–	–	–	1
*Kpn*8A3	8	*K. pneumoniae*	Faeces	145	A	75,161	JAGPOH000000000	32	R	R	R	R	S	S	S	–	–	–	1
*Kpn*8E4	8	*K. pneumoniae*	Faeces	433	E	75,162	JAGPOI000000000	4	R	R	I	R	S	S	S	I	–	–	1
*Kae*9A1	9	*K. aerogenes*	Faeces	39	A	67,827	JAGPOJ000000000	32	R	I	R	R	S	S	R	R	S	–	–
*Kpn*9A2	9	*K. pneumoniae*	Faeces	39	A	67,828	JAGPOK000000000	0.25	R	R	S	R	S	S	R	S	S	1	1
*Kpn*10A1	10	*K. pneumoniae*	Faeces	49	A	67,829	JAGPOL000000000	0.25	R	R	S	R	S	S	R	–	–	1	1
*Kpn*10A2	10	*K. pneumoniae*	Faeces	67	A	75,164	JAGPOM000000000	4	R	R	I	R	S	S	S	S	S	1	1
*Kpn*10A3	10	*K. pneumoniae*	Faeces	67	A	75,165	JAGPON000000000	16	R	R	R	R	S	S	R	R	S	–	1
*Eco*10A4	10	*E. coli*	Faeces	88	A	75,166	JAGPOO000000000	0.125	S	S	S	R	S	S	S	S	S	–	–
*Kpn*10E5	10	*K. pneumoniae*	Faeces	433	E	75,167	JAGPOP000000000	2	R	R	S	R	S	S	S	S	–	1	1

^a^ The index isolate is marked with an asterisk (*). ^b^ The day when the sample yielding the isolate was taken. The index sample date is set to day 0. ^c^ The hospital ward where the infant was cared for when the sample yielding the isolate was taken: Ward A, Ward B, Ward C, Ward D, and Ward E. ^d^ Minimum inhibitory concentration of meropenem. ^e^ Phenotypic resistance results for cefotaxime (CT), ceftazidime (CAZ), meropenem (MP), piperacillin-tazobactam (TZP), tobramycin (TM), ciprofloxacin (CI), trimethoprim/sulfamethoxazole (TS), imipenem (IP), and colistin (CO). ^f^ Phenotypic test results for ESBL and AmpC production: 1 = positive, 0 = negative, and—= not tested

### DNA extraction and whole-genome sequencing

Genomic DNA was extracted using the QIAamp DNA Mini Kit (Qiagen) for short-read sequencing and the Marmur protocol for long-read sequencing [15]. DNA quality and quantity were measured with a Nanodrop™ ND-1000 Spectrophotometer (Thermo Fisher) and a Qubit 2.0 Fluorometer (Invitrogen), respectively. The species identity was confirmed by 16S rRNA Sanger sequencing (GATC Biotech). The isolates were whole genome sequenced using either IonTorrent (isolates *Ecl1A*, *Kpn2A2*, *Kpn5B*, *Kpn6A*, *Kpn7C*, *Kpn8A1*, *Kpn9A2*, *Kpn10A1*) or Illumina (isolates *Kpn2A1*, *Kpn3A1*, *Kae3B2*, *Kpn3B3*, *Kpn3B4*, *Kae3D5*, *Kae3D6*, *Kpn4A*, *Eco8A2*, *Kpn8A3*, *Kpn8E4*, *Kae9A1*, *Kpn10A2*, *Kpn10A3*, *Eco10A4*, *Kpn10E5*) technology. The index isolate (*Ecl1A*) was also sequenced by Nanopore (full details in Supplementary material). All sequenced isolates were deposited at the Culture Collection University of Gothenburg (CCUG; Table 1).

### Sequence data analysis

The Illumina sequence data were trimmed from adapters and low-quality sequences using Trim galore! (www.bioinformatics.babraham.ac.uk/projects/trim_galore/; v. 0.4.3, parameters -stringency 3 -paired -retain_unpaired). The short-read data for all but the index isolate was assembled using SPAdes (v.3.14.1, Illumina parameters: -isolate -cov-cutoff 5; IonTorrent parameter: -iontorrent -k 21,33,55,77,99,127 -isolate -cov-cutoff 5) [16]. The short- and long-read data for the index isolate were assembled using Unicycler (v. 0.4.4, default parameters) [17] which produced complete circular assemblies of the chromosome and all plasmids. This Whole Genome Shotgun project has been deposited at DDBJ/ENA/GenBank under the accession JAGPNS000000000-JAGPOP000000000. The versions described in this paper are versions JAGPNS010000000.1- JAGPOP010000000.1 (Table 1).

The assemblies were annotated for antimicrobial resistance genes using ResFinder followed by manual inspection (v. 4.1, 90% identity, 20% coverage) [18] and the incompatibility groups of the index isolate plasmids were identified using PlasmidFinder (v. 2.1, default parameters). To investigate the transfer of the index isolate’s plasmids, the reads of the remaining isolates were mapped to the plasmid assemblies in the following way. First, the IonTorrent and the trimmed Illumina data were error-corrected using SPAdes (v 0.4.3, parameters: Illumina, -only-error-correction; IonTorrent, -only-error-correction -iontorrent) [16]. The trimmed and error-corrected paired reads were mapped to each of the five plasmids of the index isolate using BowTie2 (v. 2.3.0, default parameters) [19] and the coverage was calculated for each position using samtools (v 1.6) [20]. The complete plasmid profiles of isolates other than the index isolate were not investigated.

Epidemiological typing of all sequenced isolates was performed using MLST schemes from PubMLST.org [21]. All *K. pneumoniae* sequences were also submitted to the Institute Pasteur MLST-2.0 server and analysed with the BIGSdb database for curation and publication of core genome MLST [22]. Epidemiological strain typing of *E. coli* and *K. aerogenes* isolates was done by SNP analysis using the references *E. coli* IAI1 (accession number ENA_CU928160 and *K. aerogenes* KCTC 2190 (NC_015663.1) in CLC Genomics Workbench 12 (Qiagen; full details in Supplementary materials).

## Results

### Outbreak description

The outbreak timeline and the follow-up period are shown in Fig. 1 and Supplementary Figure S1. During the outbreak, *Enterobacteriaceae* strains producing carbapenemases of the OXA-48 group were isolated from two twin pairs and six singlets, with a mean gestational age of 27 weeks (clinical characteristics of the 10 preterm infants are shown in Supplementary Table S1). All suspected cases except the index case (Case 1) yielded a *K. pneumoniae* isolate positive for OXA-48-like carbapenemases at the time of case identification. Case 2 was identified from a positive blood culture and the others were identified by rectal screening.

The index case was identified at Ward A, where an *E. cloacae* isolate positive for the OXA-48 group (*Ecl*1A) was detected in a rectal screening culture collected on day 0 (Table 1, Fig. 1, Supplementary Fig. S1). Due to overcrowding, the patient was initially cared for in a multi-bedroom by a dedicated nurse before being transferred to a single-bed room on day 5. Since isolation routines had not been followed, rectal screening was enhanced to twice weekly from day 5, including all neonates at the ward. The index case remained on Ward A for 10 days, but no follow-up cultures were obtained. The second case was identified on day 17 on Ward A, by a blood culture yielding a *K. pneumoniae* isolate positive for the OXA-48 group (*Kpn*2A1). Rectal screening the following day detected gut colonization by similar OXA-48-producing *K. pneumoniae* in Cases 2–5. At this time point, Case 5 had been transferred to a different unit (Ward B), where Case 6 was discovered 4 days later (day 22). On day 29, Case 7 was identified at a third unit (Ward C). Three additional cases (8–10) were identified on Ward A between days 35 and 49: Cases 8 and 10 were a twin pair and Case 9 was the twin sibling of Case 3. Thus, all suspected cases except for Case 7 had a spatial link to either Ward A or Ward B and/or another case.

Screening cultures from the two twin pairs also yielded gut colonization with other enterobacterial species positive for OXA-48-like carbapenemases. *Klebsiella aerogenes* was isolated from Case 3 (*Kae*3B2) on day 28 and 11 days later from the sibling, Case 9, who, thus, harboured both *K. aerogenes* (*Kae*9A1) and *K. pneumoniae* (*Kpn*9A2) strains positive for the OXA-48 group at the time of case identification. A positive *E. coli* strain was first isolated from Case 8 (*Eco*8A2) on day 74, i.e. after the outbreak period, and 14 days later from its sibling, Case 10 (*Eco*10A4).

The infection prevention and control team identified overcrowding, deficient routine practices for milk preparation, inadequate handling of shared medical equipment, inexperienced healthcare workers, and cluttering of non-medical utensils in the patient areas as risk factors for continued transmission. Hence, control measures were focused on adherence to existing routines for infection prevention and control and included the education of both healthcare workers and parents. The extended rectal screening program was continued until day 60.

Two infants, Case 2 and Case 8, developed sepsis at 28 and 39 days of age, respectively, with blood cultures yielding *K. pneumoniae* positive for OXA-48-like carbapenemases. Of the six cases that were screened more than one year after the outbreak, three were still colonized by OXA-48-producing *Enterobacteriaceae*: two with *K. pneumoniae* (Cases 8 and 10) and one with *K. aerogenes* (Case 3). Later follow-up samples from these infants were negative (Supplementary Figure S1).

### The index isolate and its plasmids

The index isolate *E. cloacae* CCUG 67,822 (*Ecl*1A) belonged to a novel sequence type (ST), ST1584 [23]. All its plasmids, except for the smallest one (p*Ecl*A5), harboured antimicrobial resistance genes (Table 2). The *bla*_OXA-48_ gene was located as the only resistance gene on a 64 kb long IncL plasmid (p*Ecl*A2), which was near identical (two substitutions, both non-synonymous, in *traN* and *traX*) to the wide-spread OXA-48-encoding plasmid characterized by Hamprecht et al. [24]. This plasmid locates *bla*_OXA-48_ between two identical insertion sequences, IS1999, members of the transposon (Tn)1999 family [2]. The plasmid p*Ecl*A4 also carried a single resistance gene, *bla*_CMY-4_, and was almost identical (one substitution and one insertion, both in an intergenic region) to the CMY-4-encoding IncQ plasmid described by Kotsakis et al. [25]. The largest plasmid, p*Ecl*A1, carried genes encoding resistance towards β-lactams (*bla*_TEM-1_), sulphonamides (*sul2*), aminoglycosides (*aac(3)-IIa*), and fluoroquinolones (*qnrS1*). Finally, the plasmid p*Ecl*A3 harboured resistance genes towards β-lactams (*bla*_TEM-1_), sulphonamides (*sul1*) aminoglycosides (*aadA1*), trimethoprim (*dfrA15*), tetracyclines (*tet*(D)), and chloramphenicol (*catA2*; Table 2).

**Table 2:** Plasmids in the index isolate *Enterobacter cloacae* CCUG 67822 (isolate ID *Ecl*1A**)**

Plasmid ID	Size (bp)	Multiplicity ^a^	Inc group	Antimicrobial resistance genes	Closest GenBank match [%nt identity/%coverage]
p*Ecl*A1	202 089	1.88x	IncF	*bla*_TEM-1B_, *aac(3)-IIa*, *qnrS1*, *sul2*	CP058188.1 [98.84/55]
p*Ecl*A2	63 589	2.11x	IncL	*bla*_OXA-48_	LR025098.1 [100/100]
p*Ecl*A3	61 783	2.00x	IncR	*sul1*, *aadA1*, *dfrA15*, *tet*(D), *catA2*, *bla*_TEM-1D_	LR697132.1 [99.71/58]
p*Ecl*A4	6 926	9.24x	IncQ	*bla*_CMY-4_	KP205272.1 [99.94/100]
p*Ecl*A5	3 019	8.59x	–	–	CP050768.1 [100/98.54]

^a^ Plasmid multiplicity relative to the chromosome

### Detection of plasmids pEclA2 and pEclA4 in other outbreak isolates

**Fig. 2:**
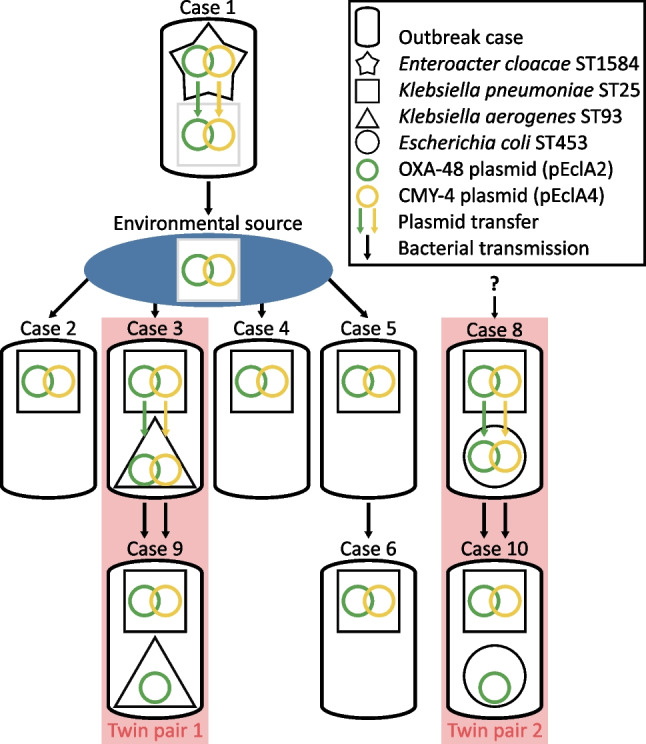
A schematic figure of the proposed bacterial transmission and plasmid transfer events during the outbreak. Grey squares represent the *K. pneumoniae* ST25 outbreak strain in sources where we have no proof of its presence

By mapping the short reads of the 23 sequenced non-index isolates onto the complete plasmid sequences of the index isolate, we could conclude that all isolates harboured a plasmid resembling p*Ecl*A2, i.e. the *bla*_OXA-48_ plasmid (Fig. 1, Tables 2 and 3). All isolates had a uniform mapping coverage over p*Ecl*A2, except for the *K. pneumoniae* isolate from Case 7 (*Kpn*7C), in which the plasmid lacked a 12 kb region (position 31 267–43 887) including, e.g. genes involved in conjugation (*mobAB* and *traHIJ*), as well as a 1.5-kb region (position 46 682–46 792) encoding a primase. Further, the plasmid of isolate *Kpn*7C did not harbour *bla*_OXA-48_ but *bla*_OXA-162_, another carbapenemase of the OXA-48 group. This isolate also lacked p*Ecl*A4, the IncQ plasmid of the index isolate, which was detected in all other *K. pneumoniae* isolates, one *K. aerogenes*, and one *E. coli* isolate (Fig. 1, Table 3, Fig. 2). None of the other plasmids of the index isolate was found in the other outbreak isolates.

**Table 3:** Molecular typing of the sequenced isolates

Isolate ID ^a^	MLST ^b^	OXA-48/ CMY-4 ^c^	Additional antimicrobial resistance genes
*Ecl*1A*	ST1584	+ / +	*bla*_CMH-3-like_, *fosA*, *aadA1*, *ere*(A), *bla*_TEM-1B_, *aac(3)-IIa*, *qnrS1*, *sul2*, *sul1*, *aadA1*, *dfrA15*, *tet*(D), *catA2*, *bla*_TEM-1D_
*Kpn*2A1	ST25	+ / +	*fosA*, *bla*_SHV-11_, *dfrA12*, *aadA2*, *sul1*, *mph*(A)
*Kpn*2A2	ST25	+ / +	*fosA*, *bla*_SHV-11_, *dfrA12*, *aadA2*, *sul1*, *mph*(A)
*Kpn*3A1	ST25	+ / +	*fosA*, *bla*_SHV-11_, *dfrA12*, *aadA2*, *sul1*, *mph*(A)
*Kae*3B2	ST93	+ / -	*fosA*
*Kpn*3B3	ST25	+ / +	*fosA*, *bla*_SHV-11_, *dfrA12*, *aadA2*, *sul1*
*Kpn*3B4	ST25	+ / +	*fosA*, *bla*_SHV-11_, *dfrA12*, *aadA2*, *sul1*
*Kae*3D5	ST93	+ / -	*fosA*
*Kae*3D6	ST93	+ / +	*fosA*
*Kpn*4A	ST25	+ / +	*fosA*, *bla*_SHV-11_, *dfrA12*, *aadA2*, *sul1*, *mph*(A), *sul2*, *catA1*, *tet*(D)
*Kpn*5B	ST25	+ / +	*fosA*, *bla*_SHV-11_, *dfrA12*, *aadA2*, *sul1*, *mph*(A), *sul2*, *catA1*, *tet*(D)
*Kpn*6A	ST25	+ / +	*fosA*, *bla*_SHV-11_
*Kpn*7C	ST37	- / -	*fosA*, *bla*_SHV-11_, *bla*_CTX-M-15_, *bla*_OXA-162_
*Kpn*8A1	ST25	+ / +	*fosA*, *bla*_SHV-11_, *dfrA12*, *aadA2*, *sul1*, *mph*(A), *sul2*, *catA1*, *tet*(D)
*Eco*8A2	ST453	+ / +	*bla*_TEM-1B_, *tet*(A)
*Kpn*8A3	ST25	+ / +	*fosA*, *bla*_SHV-11_
*Kpn*8E4	ST25	+ / +	*fosA*, *bla*_SHV-11_
*Kae*9A1	ST93	+ / -	*fosA*
*Kpn*9A2	ST25	+ / +	*fosA*, *bla*_SHV-11_, *dfrA12*, *aadA2*, *sul1*, *mph*(A)
*Kpn*10A1	ST25	+ / +	*fosA*, *bla*_SHV-11_, *dfrA12*, *aadA2*, *sul1*, *mph*(A), *sul2*, *catA1*
*Kpn*10A2	ST25	+ / +	*fosA*, *bla*_SHV-11_
*Kpn*10A3	ST25	+ / +	*fosA*, *bla*_SHV-11_, *dfrA12*, *aadA2*, *sul1*, *mph*(A), *sul2*, *catA1*, *tet*(D)
*Eco*10A4	ST453	+ / -	*bla*_TEM-1B_, *tet*(A)
*Kpn*10E5	ST25	+ / +	*fosA*, *bla*_SHV-11_

^a^ The index isolate is marked with an asterisk (*). ^b^ Multi-locus sequence type. ^c^ Carriage of the plasmids encoding OXA-48 and CMY-4, p*Ecl*1A2 and p*Ecl*1A4, respectively

### Epidemiological strain typing

The MLST analysis revealed that all *K. pneumoniae* isolates belonged to ST25 except for isolate *Kpn*7C (Case 7) which belonged to ST37 (Table 3). Further analyses using core genome MLST (573 loci) showed no more than two allele differences between the ST25 isolates, strongly indicating strain identity. Isolate *Kpn*7C clearly belonged to a separate strain as it differed in at least 475 core alleles when compared to the ST25 isolates. Based on these data, and the fact that the *bla*_OXA-162_ plasmid of isolate *Kpn*7C did not fully resemble p*Ecl*A2, we could conclude that Case 7 was not part of the outbreak.

The four *K. aerogenes* isolates (*Kae*3B2, *Kae*3D5, *Kae*3D6, and *Kae*9A1) from twin pair 1, Case 3 and 9, belonged to ST93 (Table 3), and the SNP analysis of these isolates identified 1–7 SNPs difference using *K. aerogenes* KCTC 2190 as the reference and 0–4 SNPs using *Kae*3D5 as the reference, demonstrating that the four isolates belonged to the same strain. Finally, the two *E. coli* isolates (*Eco*8A2 and *Eco*10A4) from twin pair 2, Cases 8 and 10, both belonged to ST453 (Table 3), and the SNP analysis did not reveal any true SNPs when comparing the two isolates.

## Discussion

In this study, we describe in detail an outbreak caused by OXA-48-producing *Enterobacteriaceae*, involving nine preterm infants at a Swedish NICU. To the best of our knowledge, this is the first description of an outbreak of OXA-48-producing *Enterobacteriaceae* in a neonatal setting in northern Europe.

Interestingly, all cases identified in the present outbreak were preterm infants (gestational age at birth 24–34 weeks). Both prematurity per se, but also increased exposure to antibiotics in this group may hamper colonization by protective anaerobic gut bacteria [10]. This predisposes to overgrowth by *Enterobacteriaceae*, and high population counts of these bacteria might promote bacterial conjugation and horizontal gene transfer [14, 26].

The extended rectal screening program, initiated after the identification of the index case, ensured early detection of each new case and in-depth molecular data was obtained using whole-genome sequencing. The approach of combining genomic and clinical epidemiological data allowed us to create a likely scenario for the plasmid transfer and strain dissemination (Fig. 2).

Thus, we propose that the *E. cloacae* index strain transferred two plasmids, p*Ecl*A2 and p*Ecl*A4, to the outbreak strain *K. pneumoniae* ST25. This likely occurred in the gut of Case 1, who was identified as both the index and the primary case in this outbreak. However, since Case 1 was excluded from further screening after the isolation of the *E. cloacae* strain, carriage of *K. pneumoniae* ST25 could not be established for this patient. Therefore, an unidentified carrier in whom the plasmid transfer occurred, either from *E. cloacae* ST1584 to *K. pneumoniae* ST25 or the reverse, cannot be excluded, although the meticulous rectal screening program reduces the likelihood of an undetected neonatal case within Ward A. It is also unlikely that the plasmid transfer occurred outside a human host since high bacterial densities are required for conjugation [26].

This outbreak lasted for 50 days; hence, multiple incidents would have contributed to the continued spread of the *K. pneumoniae* ST25 strain. However, the simultaneous identification of Cases 2–5, after several negative screening cultures, indicates that they obtained the *K. pneumoniae* ST25 strain at approximately the same time, although the different resistance profiles of their isolates could imply several sources of transmission (Table 2). As Case 1 had been absent from the ward for 7 days at the time of their identification, we hypothesize that *K. pneumoniae* ST25 spread from Case 1 to Cases 2–5 through one or several contaminated fomites within Ward A. This could not be further investigated as no environmental cultures were collected during the outbreak. However, the isolation of outbreak clones of *K. pneumoniae* from the NICU environment has been reported in several studies [7, 27–31].

Within Ward B, *K. pneumoniae* ST25 was likely transmitted from Cases 5 to Case 6 since there was a clear spatial and temporal link between these cases. The source and route of spread to Case 8 were obscure, while the discovery of Cases 9 and 10 was expected due to the spread from their respective twin sibling.

The genetic analysis of isolate *Kpn*7C separated Case 7 from the outbreak. This illustrates the potential clinical benefits of access to rapid epidemiological genetic typing in future outbreak investigations, as unnecessary resource-consuming investigations may be avoided at an early stage of an outbreak.

Although we could only speculate on when and where the plasmids p*Ecl*A2 and p*Ecl*A4 have transferred between the index *E. cloacae* strain and the *K. pneumoniae* outbreak strain, other plasmid transfer events were more clearly indicated. Based on the genetic analysis and timeline of follow-up isolates from the two twin pairs, in vivo horizontal transfer of the plasmids p*Ecl*A2 and p*Ecl*A4 from the outbreak *K. pneumoniae* strain to other enterobacterial species was strongly suggested (Fig. 1). We hypothesize that an initial transfer of p*Ecl*A2, followed by p*Ecl*A4 from *K. pneumoniae* ST25 to the *K. aerogenes* ST93 strain occurred in the gut of Case 3, and that both the *K. pneumoniae* ST25 strain (with both plasmids) and the *K. aerogenes* ST93 strain (with plasmid p*Ecl*A2) were transmitted to its sibling, Case 9. Similarly, we propose that p*Ecl*A2 and p*Ecl*A4 were transferred from the *K. pneumoniae* ST25 strain to the *E. coli* ST453 strain in the gut of Case 8. Somewhat later the *E. coli* ST453 strain carrying only the p*Ecl*A2 plasmid was isolated from its twin sibling, Case 10. This could either be a result of the transfer of p*Ecl*A2 from *K. pneumoniae* ST25 to *E. coli* ST453 in the gut of Case 10, or the spread of an *E. coli* ST453 variant carrying only p*Ecl*A2 from Cases 8 to Case 10.

Our data, therefore, give strong indications, but no proof of plasmid transfer. However, transfer of resistance plasmids in vivo between *E. coli* strains in the gut microbiota of infants has previously been demonstrated [13, 32], and in vitro conjugation experiments have shown horizontal transfer of carbapenemase-encoding outbreak plasmids between gut bacteria [24, 33, 34]. Further, a recent experimental study suggests a high potential for horizontal gene transfer in the gut microbiota of preterm neonates [14].

In summary, strain typing and plasmid tracing using whole-genome sequencing in combination with clinical epidemiology data proved to be a useful tool for the analysis of the transmission of bacterial strains and in vivo transfer of plasmids during this outbreak. Our methods allowed the exclusion of a strain producing an OXA-48-like carbapenemase unrelated to the outbreak, while several enterobacterial strains of different species could be identified as part of the outbreak, due to their carriage of identical OXA-48-encoding plasmids. Although rapid less discriminating diagnostic methods are crucial for an early outbreak response, the ability to discriminate non-related cases at an early stage is important for effective resource allocation. Even more so in high-prevalence settings where incidental findings of carbapenemase-producing *Enterobacteriaceae* are common.

## Supplementary Information

Below is the link to the electronic supplementary material.

Supplementary file1 (DOCX 343 KB)

## Data Availability

The genome assemblies of the included isolates have been submitted til NCBI Assembly. The accession numbers for the assemblies are listed in Table 1 together with additional information on the included isolates.
