# Supplementary material for: Outbreak of OXA-48-producing Enterobacteriaceae in a neonatal intensive care unit in Western Sweden
Source: Eur J Clin Microbiol Infect Dis. 2023 Mar 20;42(5):597–605. doi: 10.1007/s10096-023-04584-y (PMC10105658; doi:10.1007/s10096-023-04584-y)
Supplement: Supplementary file 1 — Supplementary file1 (DOCX 343 KB) [file 10096_2023_4584_MOESM1_ESM.docx]

Supplementary Material

**Outbreak of OXA-48-producing Enterobacteriaceae in a neonatal intensive care unit in Western Sweden**

European Journal of Clinical Microbiology and Infectious Diseases

Erika Tång Hallbäck*, Anna Johnning, Sofia Myhrman, Marie Studahl, Elisabet Hentz, Anders Elfvin and Ingegerd Adlerberth

*Department of Infectious Diseases, Institute of Biomedicine, Sahlgrenska Academy, University of Gothenburg, Gothenburg, Sweden. [erika.hallback@gu.se](mailto:erika.hallback@gu.se)

# Bacterial identification, antimicrobial susceptibility testing, and identification of resistance determinants

At the University Hospital Clinical Microbiology laboratory, screening samples and clinical samples were handled and analyzed according to standardized laboratory protocols used in clinical routine at the time of the outbreak. Briefly, rectal swab screening samples (ESwab COPAN Diagnostics inc.) were cultured overnight on Drigalski agar plates with one cefuroxime disk (CXM30) and one ertapenem disk (ETP10) firmly applied on the agar surface. Isolates showing reduced sensitivity to either cefuroxime or ertapenem were analyzed further. Species identity was determined using MALDI-TOF (VITEK MS, bioMérieux), and phenotypic antibiotic susceptibility testing was performed according to EUCAST guidelines ( http://www.eucast.org/clinical_breakpoints). According to Nordicast guidelines (<http://www.nordicast.org/>), isolates of relevant species showing resistance to cephalosporins were tested for ESBL and AmpC production using the double-disk synergy test [13] and the cloxacillin synergy test [14], respectively. Isolates yielding a zone diameter < 27 mm for meropenem (MP10) and/or a meropenem MIC >0.125 mg/L were tested for carbapenemase production using the Rosco Diagnostica KPC and MBL Confirm Kit (Rosco Diagnostica, Taastrup, Denmark), followed by a real-time PCR detecting genes for the OXA-48, NDM, KPC, VIM, and GES groups of carbapenemases [15]. Repeated isolates with meropenem MIC >0.125 mg/L from a known case were confirmed by PCR only if they differed from previous isolates regarding species identity, phenotypic resistance profile, results of the Rosco test, or if several weeks had passed since the last sampling.

# Details of whole-genome sequencing

IonTorrent sequencing was performed at the Clinical Microbiology Laboratory, Sahlgrenska, Sweden. DNA libraries (300 bp) were prepared with the IonXpress™ Plus Fragment Library Kit on an AB Library Builder™ System (ThermoFisher Scientific). Fragment size analysis was done with a 4200 TapeStation (Agilent Technologies) followed by Agencourt AMPure XP magnetic beads purification (Beckman Coulter). Illumina sequencing libraries were made using the TruSeq DNA Nano Library Prep Kit (Illumina) and sequenced on a MiSeq™ System (2x300bp) (Illumina) at Clinical Genomics Gothenburg, SciLife Labs, Sweden. Long-read sequencing of the index isolate (*Ecl1A*) was performed at the Culture Collection University Gothenburg (CCUG) laboratories on a MinION sequencer (Oxford Nanopore Technologies), using a flow cell model FLO-MIN 106 version R9.4.1 and the MinION software (Oxford Nanopore Technologies; version 18.12.9 with default parameters).

# SNP-analysis details

To improve the read mapping, local realignment was used (mapping parameters: match score 1, mismatch cost 2, and a linear gap cost for insertions/deletions 3; variant calling parameters: minimum coverage 20, minimum count 10, minimum frequency 20 %). A noise filter was used to take the quality scores of the surrounding bases into account (parameters: neighbourhood radius 5 bp, minimum neighbourhood quality 15). Reads shorter than 20 base pairs were filtered out and a neighbour-joining algorithm was used for the SNP analysis (parameters: minimum coverage required in each sample 20, minimum coverage percentage of average required 10, prune distance 15, minimum z-score required 1.96).

**
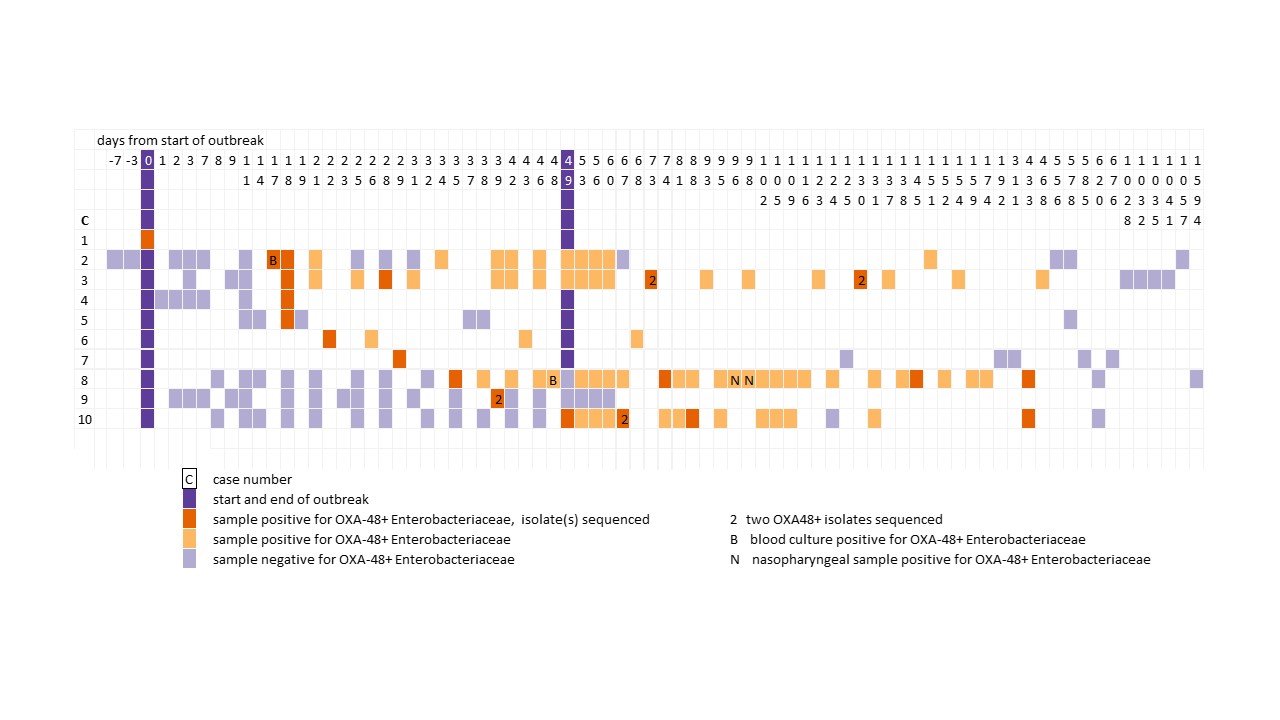
**

**Figure S1.** Rectal screening samples (n=155) obtained from the start of the outbreak from the ten suspected cases and the four clinical samples from these infants (two blood cultures and two nasopharyngeal samples) which yielded Enterobacteriaceae isolates assessed as positive for carbapenemases of the OXA-48 group. Negative clinical samples are not shown. Colours are used to indicate samples positive (orange) or negative (lilac) for Enterobacteriaceae with carbapenemases of the OXA-48 group, and samples from which isolates were selected for whole-genome sequencing are highlighted (dark orange). From four samples, two different Enterobacteriaceae isolates were selected for sequencing, and one isolate was selected from each of the remaining 16 samples. In total, 24 isolates were selected for sequencing.

**Table S1.** Clinical characteristics of the ten suspected cases involved in the outbreak with OXA-48-producing Enterobacteriaceae in an NICU of a tertiary care University Hospital in Western Sweden.

| **Clinical characteristics of the suspected outbreak cases** | N (%) or median (range) |
| --- | --- |
| Number of infants | 10 |
| Girls | 4 (40%) |
| Gestational age at birth | 28.9 weeks (24.3 – 34.6) |
| Birth weight | 1075 g (555 – 2600) |
| Age at first positive culture with OXA-48+ bacteria | 20 days (1 – 45) |
| Treated with antibiotics in the first week of life | 10 (100%) |
| Days on antibiotics in the first week of life | 7 days (3.5 – 7) |

All cases were given antibiotics during their first week of life and all but two cases were treated with antibiotics for seven days during week one. Also, all the included cases were preterm born between gestational weeks 24 and 34. During the half-year covering the outbreak in 2015 – quarter 2 to quarter 3 – the proportion of infants born before gestational week 36 at the NICU was 51% (272 out of 534) and the proportion of infants born before gestational week 28 was 9% (50 out of 534).


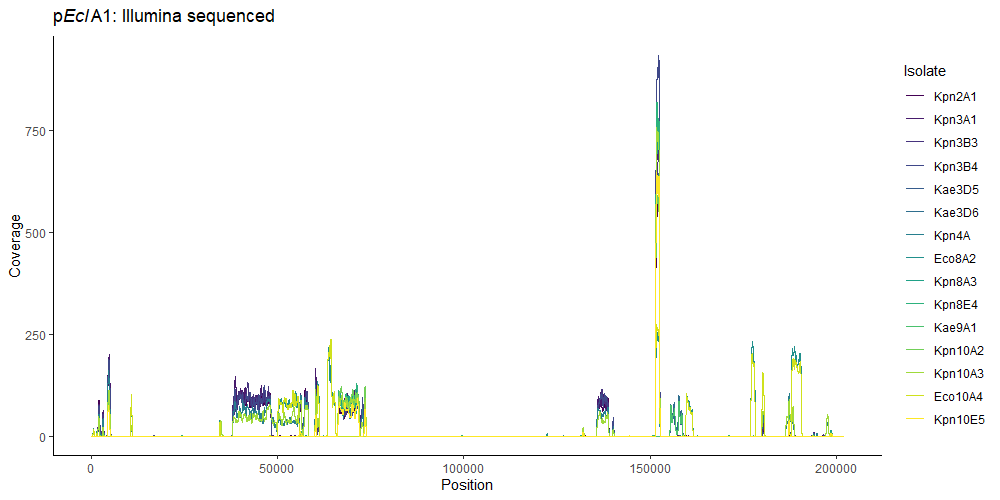


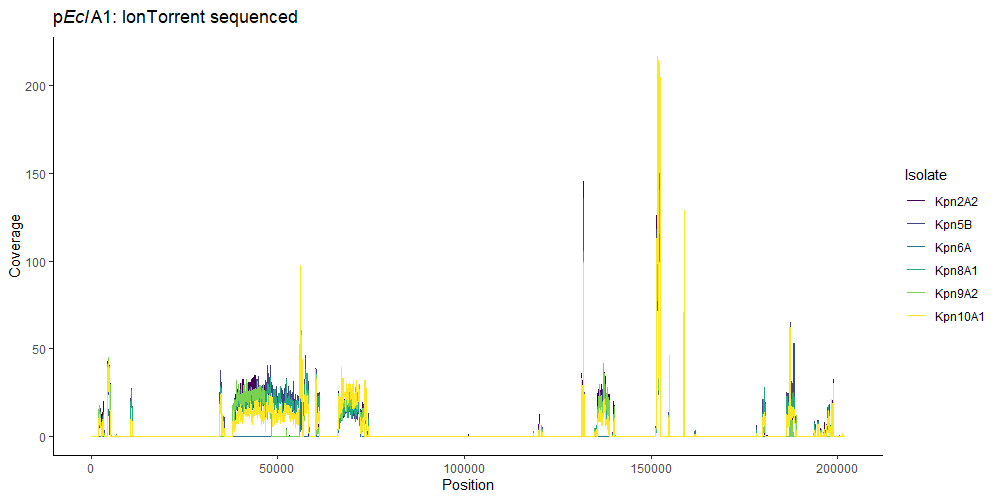


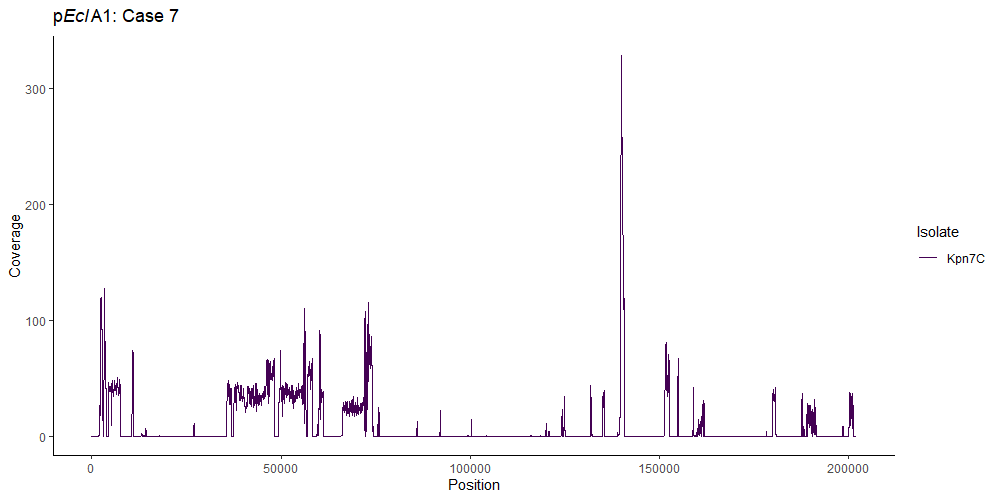


**Figure S2.** a) Coverage plots (rolling average over 100 bp) for the plasmid p*Ecl*A1 from the index isolate.


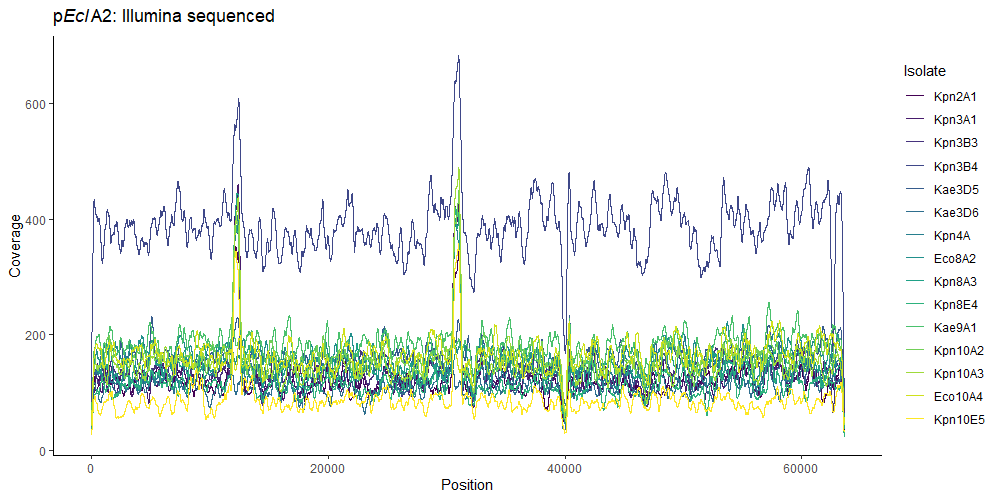


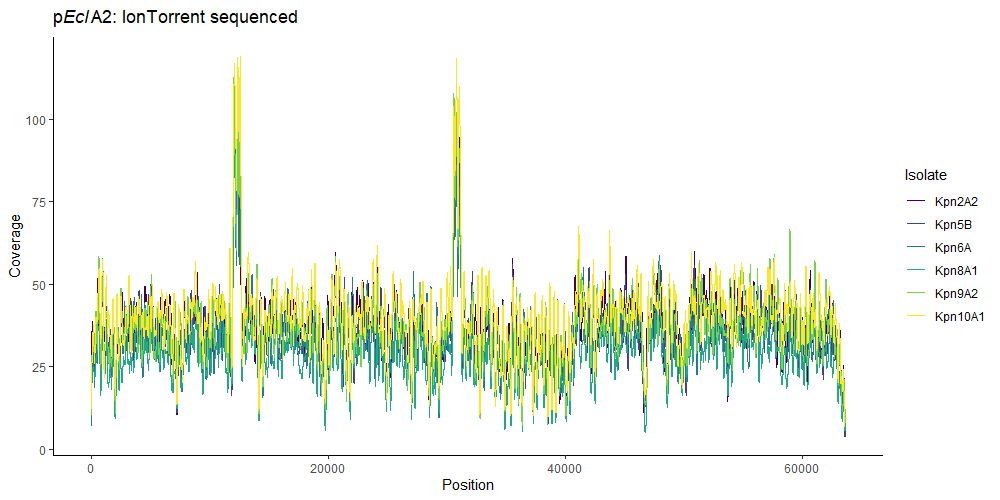


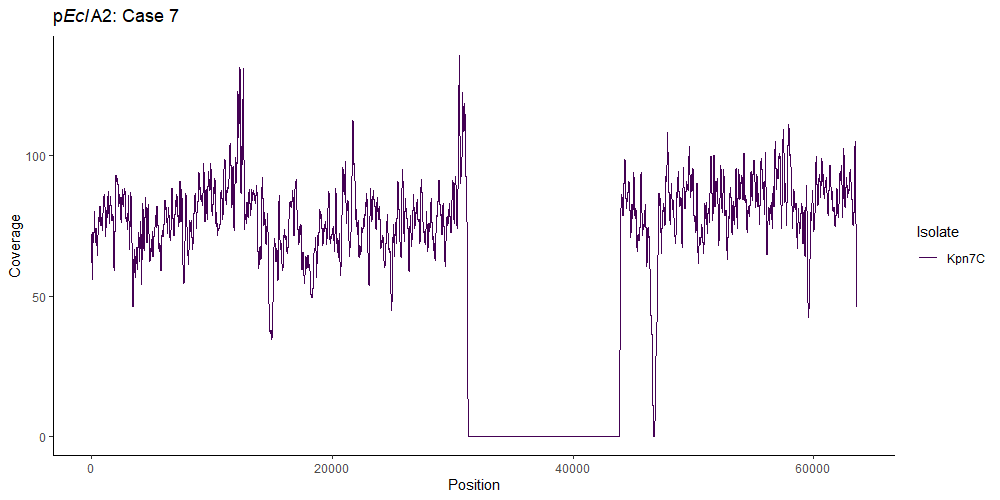


**Figure S2.** b) Coverage plots (rolling average over 100 bp) for the plasmid p*Ecl*A2 from the index isolate.


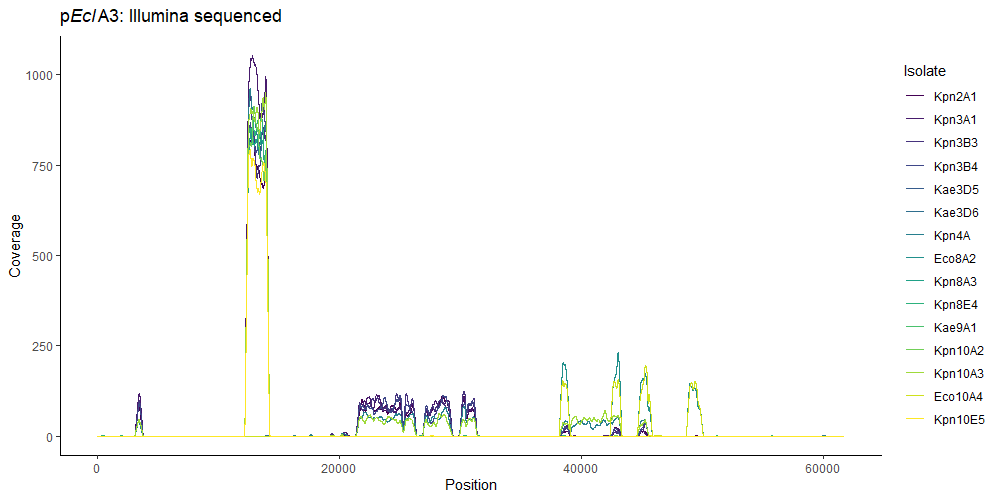


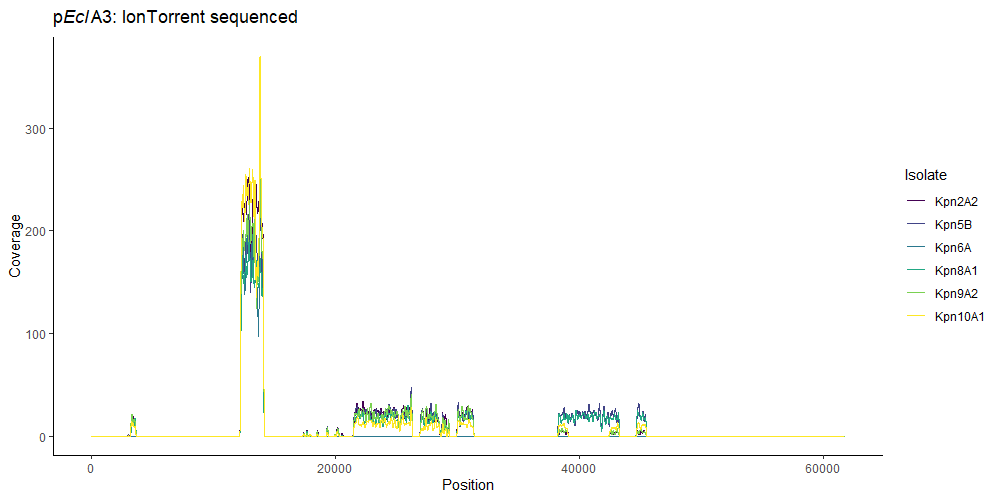


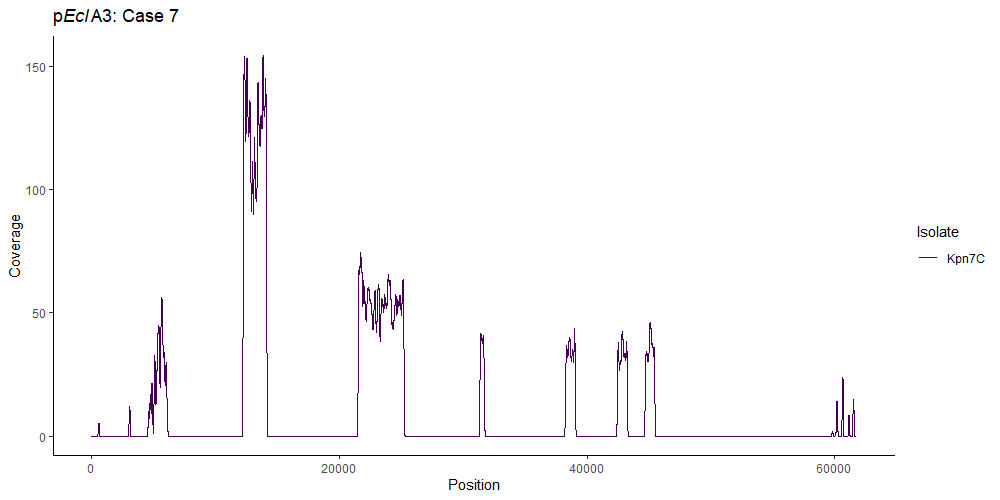
**Figure S2.** c) Coverage plots (rolling average over 100 bp) for the plasmid p*Ecl*A3 from the index isolate.


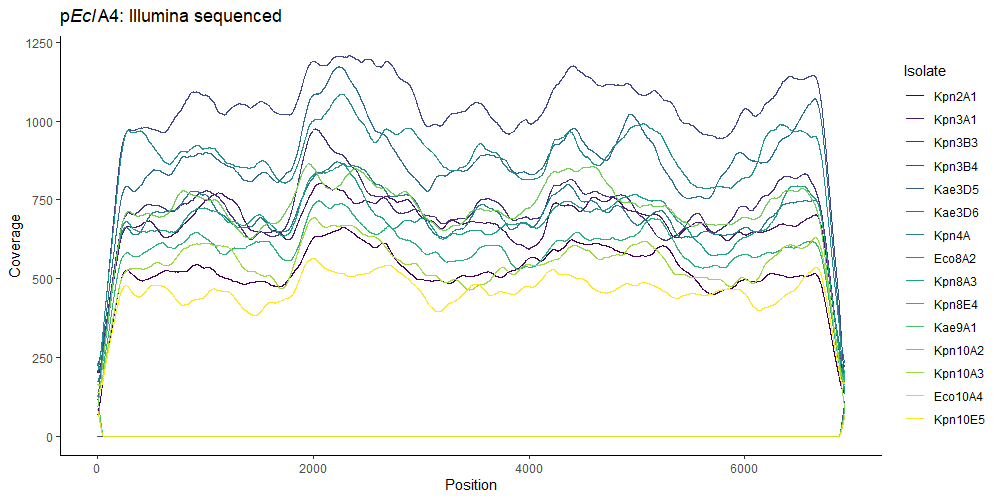


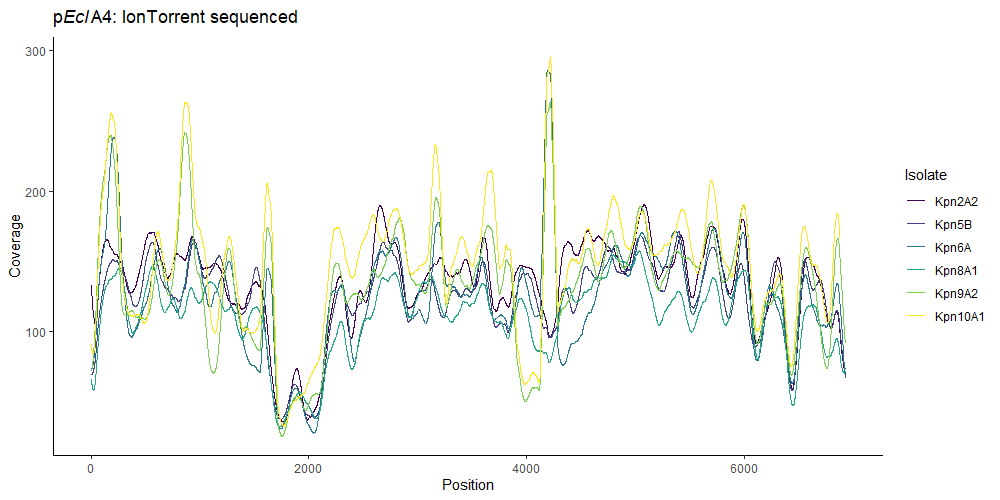


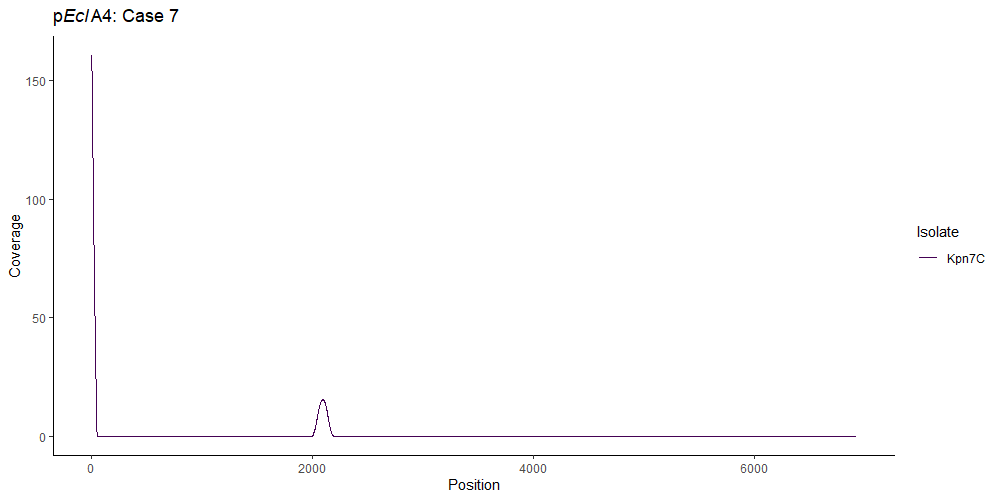


**Figure S2.** d) Coverage plots (rolling average over 100 bp) for the plasmid p*Ecl*A4 from the index isolate.


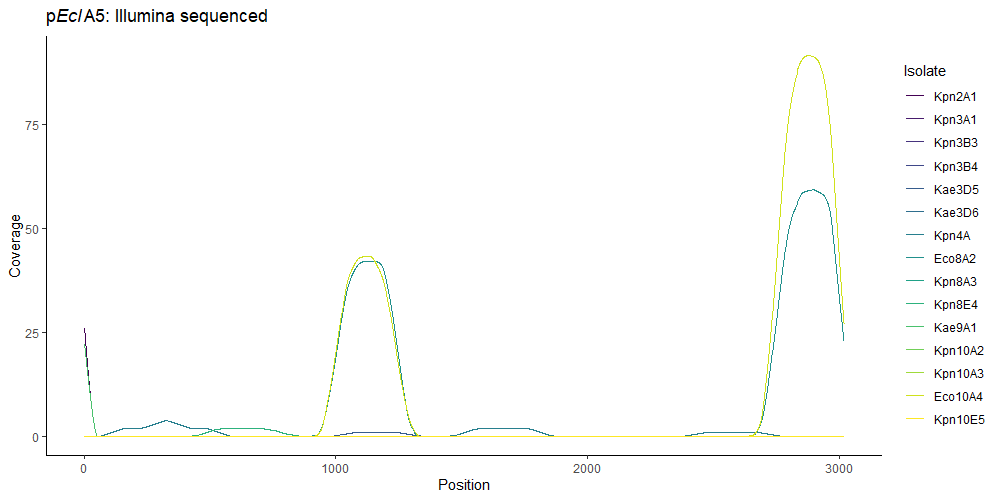


**Figure S2.** e) Coverage plots (rolling average over 100 bp) for the plasmid p*Ecl*A5 from the index isolate. The remaining sequenced isolates had zero coverage over the entire plasmid p*Ecl*A5.
